# Progress in the genetics of uveitis

**DOI:** 10.1038/s41435-022-00168-6

**Published:** 2022-04-04

**Authors:** Xiu-Feng Huang, Matthew A. Brown

**Affiliations:** 1 The Second Affiliated Hospital of Wenzhou Medical University, Wenzhou, Zhejiang China; 2 Queensland University of Technology, Centre for Genomics and Personalised Health, School of Biomedical Sciences, Faculty of Health, Translational Research Institute, Woolloongabba, Qld Australia; 3 Department of Medical and Molecular Genetics, Faculty of Life Sciences and Medicine, King’s College London, London, England; 4 Genomics England, London, UK

**Keywords:** Disease genetics, Functional genomics

## Abstract

Uveitis is the most common form of intraocular inflammatory disease and is a significant cause of visual impairment worldwide. Aetiologically, uveitis can also be classified into infectious uveitis and non-infectious uveitis. The common non-infectious forms of uveitis include acute anterior uveitis (AAU), Behçet’s disease (BD), Vogt-Koyanagi-Harada (VKH) disease, birdshot chorioretinopathy (BSCR), sarcoid uveitis. In addition, a few monogenic autoinflammatory disorders can also cause uveitis, such as Blau Syndrome and haploinsufficiency of A20 (HA20). Although the exact pathogenesis of non-infectious uveitis is still unclear, it is well-recognised that it involves both genetic and environmental risk factors. A hallmark of uveitis is its strong associations with human leucocyte antigens (HLA). For examples, AAU, BD and BSCR are strongly associated with HLA-B27, HLA-B51, and HLA-A29, respectively. In uveitis studies, multiple GWAS have successfully been conducted and led to identification of novel susceptibility loci, for example, *IL23R* has been identified in BD, VKH and AAU. In this review, we summarize the latest progress on the genetic associations of both HLA and non-HLA genes with major forms of uveitis, including AAU, BD, VKH, BSCR, sarcoid uveitis, Blau Syndrome and HA20, and potential future research directions.

## Introduction

Uveitis is the most common form of intraocular inflammatory disease, leading to 5–10% of visual impairment worldwide [1]. Uveitis can be anatomically classified into anterior uveitis, intermediate uveitis, posterior uveitis, and panuveitis. In addition, according to aetiological factors, uveitis can also be classified into infectious uveitis and non-infectious uveitis. Of the two categories, non-infectious uveitis is the most commonly seen and important type, and includes acute anterior uveitis (AAU), Behçet’s disease (BD), and Vogt-Koyanagi-Harada (VKH) disease [2]. Although the exact pathogenesis is still unclear in many forms of uveitis, it is well-recognised that non-infectious uveitis involves both genetic predisposition and environmental risk factors [3].

Strong associations of human leucocyte antigens (HLA) with different forms of uveitis have been identified since the early 1970s [4]. With advances in sequencing and genotyping methodology, genetic studies have enhanced our understanding of the disease aetiology. Over the past decade, genome-wide association study (GWAS) in particular has proven to be a powerful and robust method to identify genetic associations with complex diseases, particularly immune-mediated diseases [5]. The discoveries of novel associations between genomic variants and diseases not only has provided new insights into biological pathways [5], but can also lead to clinical translational applications in diagnosis, prevention, and treatment. This review will summarize the latest progress on the genetic associations of HLA and non-HLA genes with major forms of uveitis, including AAU, BD, VKH, birdshot chorioretinopathy, sarcoid uveitis, and Blau Syndrome.

## Acute anterior uveitis

Acute anterior uveitis (AAU) is the most common type of uveitis, characterized by episodic, typically unilateral, sudden-onset, inflammation of the iris and/or ciliary body. Its clinical presentations encompass pain, eye redness, photophobia, and blurred vision, associated with cellular infiltration and fibrin formation in the anterior chamber (AC). Episodes last a mean of 6–8 weeks [6]. Recurrent episodes can lead to synechiae formation, glaucoma, and visual impairment/loss. AAU is frequently associated with spondyloarthropathies, such as ankylosing spondylitis (AS), psoriatic arthritis, and inflammatory bowel disease [7]. For example, Wang et al. investigated a total of 202 patients presenting with AAU who underwent clinical and radiographic (CT) screening for sacroiliitis, and found that 80 (39.6%) had AS [8].

Multiple lines of evidence from humans indicate that genetic components play a critical role in AAU. Derhaag et al. found that the prevalence of AAU in HLA-B27-positive first-degree relatives of AAU patients was 13%, significantly higher than the frequency of 1% in the HLA-B27-positive individuals without affected relatives, indicating high familiality [9]. Recently, our group’s GWAS for AAU demonstrates that AAU is a highly heritably disorder [10]. The contribution of common genetic variants to overall disease risk (‘common variant heritability’) was ~0.4 in the comparison between AS patients with AAU and AS patients without AAU [10]. Interestingly, in the comparison between AS patients with AAU and controls, the estimated heritability reached 0.7 [10].

AAU is strongly associated with human leucocyte antigen (HLA)-B27. The association between HLA-B27 and AAU was originally reported in 1973 and remains one of the strongest human HLA-disease associations [4]. The prevalence of HLA-B27 among AAU patients reaches 80% [8], whereas it is only ~6% in general populations [11]. In 2015, Robinson et al. reported the prevalence of HLA-B27 was 81.8% (613 of 749 patients) in the group with AS and ophthalmologist-diagnosed AAU and 92.0% (633 of 688 patients) in the group with AS and self-reported AAU [12]. Another larger genetic study reported the frequency of HLA-B27 in the AS patients with AAU was 92.7% (1307/1409), and the prevalence was 76.4% (1296/1695) in the cohort of AS patients without AAU [13]. A recent analysis of British patients receiving biologic medications for AS found that 23.5% of patients reported AAU, and that HLA-B27 was associated with an increased risk of AAU complicating AS (HLA-B27 carriage in overall cohort 80.1%, in those with AS and AAU, 88.7%) [14].

Whilst the strongest genetic association of AAU is with HLA-B27, there is strong evidence for the involvement of other HLA alleles with AAU. The development of methods by which HLA alleles can be accurately imputed from SNP microarray data has greatly facilitated this research, providing a low cost and highly accurate HLA typing method enabling large sample sizes to be studied, and at the same time, for variation in ethnicity (‘population stratification’) to be assessed and controlled for. Using the Illumina Immunochip Infinium microarray [15], Robinson et al. conducted genetic analysis between AAU and healthy control subjects found significant association over *HLA-B*, corresponding to the *HLA-B27* tag SNP rs116488202 [12]. Further analysis showed that HLA-B27 homozygotes were at increased risk compared with heterozygotes (odds ratio 1.8, 95% CI 1.3–2.2). Another study conducted by comparing AS patients with AAU versus healthy controls demonstrates that *HLA-B27* and HLA-A*0201 were strongly associated with AAU using Illumina Exomechip microarray [13]. A large direct genotyping study confirmed the association of HLA-B27 with an increased risk of AAU amongst AS patients, including in African ancestry patients, and suggested a protective association of HLA-B*08 for AAU amongst AS cases (odds ratio 0.48, *P* = 10^−4^) [16]. Whether these non-HLA-B alleles are directly associated with AS or reflect association of other major histocompatibility complex genes is unclear at this point.

In addition to *HLA-B*, SNP microarray studies have reported the association of 3 non-MHC loci with genome-wide significance (*P* < 5 × 10^−8^), including *ERAP1*, *IL23R* and one intergenic region (2p15). Suggestive level of significance (*P* < 5 × 10^−6^) was also been observed with SNPs in five additional loci, including *IL10-IL19*, *IL18R1-IL1R1*, *IL6R*, 1q32, and a retinal-related gene *EYS* [12]. Candidate-gene association studies have reported nominal SNP associations in Interleukins (ILs) genes and tumour necrosis factor (TNF) genes correlated with anterior uveitis [17–19]. Another key component is the complement system, which is also involved in innate immunity and inflammatory responses. Genetic associations of complement factor H (CFH), complement factor B (CFB), complement factor I (CFI) and complement factor H related 2 (CFHR2) have been reported [8, 20–23]. Notably, CFB localizes to the MHC class III region. These complement genes and the TNF gene are in strong linkage disequilibrium with HLA-B27, and their association is likely to have been substantially if not completely driven by HLA-B27 carriage differences between cases and controls. As with most candidate gene association studies, the analysis for these studies did not control for potential population stratification effects either, and thus differences in the ethnic makeup of cases and controls studied could have affected the results.

Recently, our group applied GWAS to characterise the genetic associations of AAU, on the basis of comparing 2752 AS patients with AAU versus 3,836 AS patients without AAU. This study identified one locus associated with AAU at genome-wide significance, rs9378248, lying close to *HLA-B*. HLA imputation demonstrated HLA-B*27 (*P* = 1.86 × 10^−42^) was the most significantly associated allele with AAU. Suggestive association (*P* > 5 × 10^−8^ but < 1 × 10^−5^) was observed at eleven additional loci, including previously reported AS loci *ERAP1* (rs27529) and *NOS2* (rs2274894). In addition to these discoveries, association was seen at loci not previously known to be associated with AAU or AS, including *MERTK*, *KIFAP3*, *CLCN7*, *ACAA2* and five intergenic loci [10]. The summary data-based Mendelian randomization (SMR) for AAU with eQTL was performed to determine the most likely causal genes at associated loci. Interestingly, the most significant signals were *ERAP1* and *MERTK*. These findings suggest that *ERAP1* and *MERTK* are the most functionally relevant genes among those loci [10].

**Fig. 1:**
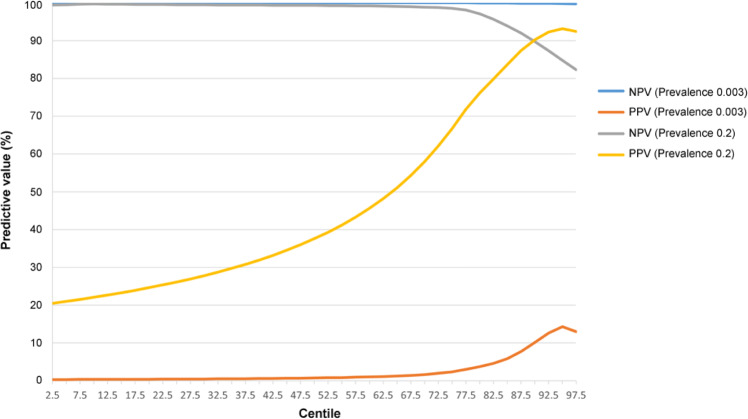
Positive (PPV) and negative predictive values (NPV) for patients with AS with AAU for centiles of genetic risk scores [10]. The prevalence of patients with both AS and AAU is assumed to be 0.3% among the general population, and 20% in AS patients attending rheumatology outpatient clinics.

**Fig. 2:**
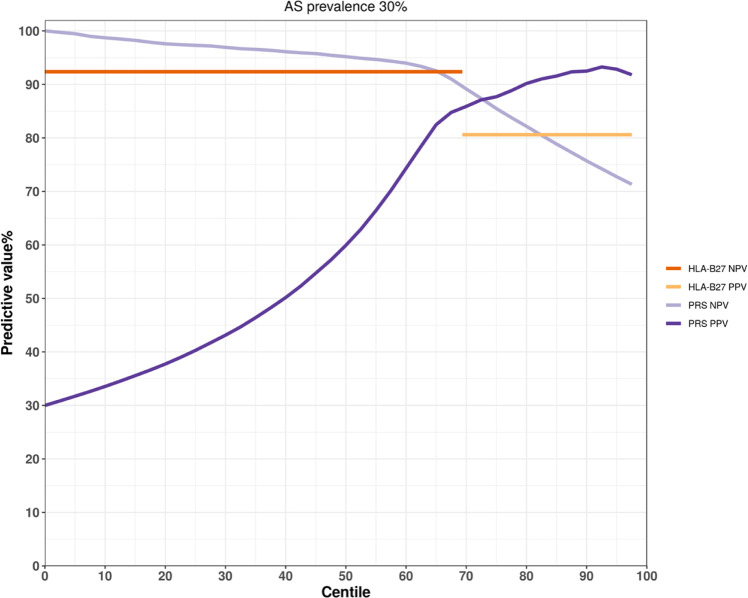
Positive (PPV) and negative predictive values (NPV) of PRS and HLA-B27 for AS [24]. Positive (PPV) and negative predictive values (NPV) of Polygenic Risk Scores (PRS) and HLA-B27 for ankylosing spondylitis (AS). The prevalence of AS is assumed to be 30%, the likely prevalence of AS amongst patients <45 years of age attending outpatient services with chronic back pain lasting >3 months.

Our analysis for the first time showed that genome-wide polygenic risk scores (PRS) have strong power in identifying individuals at high risk of either AS with AAU or AS alone. In the comparison of patients with AS with AAU (AS + AAU+) versus controls, genome-wide PRS has higher discriminatory capacity (AUC = 0.96) than computing PRS using HLA-B27 alone (AUC = 0.92) [10]. Assuming the prevalence of AS + AAU+ cases is 0.3% among the general population, individuals in the top 10% of genetic risk had an estimated genetic risk of developing AS with AAU of 10.1%, and those in the bottom 85% had <0.1% chance of developing the disease (Fig. 1) [10]. Considering the situation in outpatient clinics and assuming the prevalence is 20%, those in the top 10% and top 5% of genetic risk had an estimated genetic risk of developing disease of 90.3% for 93.2%, respectively (Fig. 1) [10]. Similar results were also reported in PRS study of AS cases. In individuals of European descent, PRS had better discriminatory capacity (AUC = 0.924) better than for HLA-B27 testing alone (AUC = 0.869) or MRI (AUC = 0.885) [24]. Assuming a prior probability for AS of 30% in clinical practice, Li et al reported the PRS PPV is >80.6% for top 35% of AS cases, showing a higher maximum value (93.3%) than does HLA-B27 (80.6%) (Fig. 2). The PRS NPV is >92.4% for 65% of cases, with a higher maximum value (99.6%) than does HLA-B27 (92.4%) (Fig. 2). These findings suggested that PRS could assist clinical diagnosis.

A key challenge in these studies is distinguishing associations of AAU as opposed to of AS. This has largely been achieved using AS cases who do not yet have AAU as controls. This model though has two significant weaknesses. Firstly, whilst it is effective at identifying AAU associations over and above any association the locus may have with AS, it cannot identify associations of AAU where the association has similar strength to any association the locus may have with AS. In that setting, no association is seen in the comparison of AS with AAU compared with AS without AAU, despite the locus being associated with AAU. To identify such associations, GWAS of AAU cases that lack concomitant AS will be required. Secondly, as many of the AS cases without AAU will ultimately develop AAU, inevitably some misclassification occurs, resulting in loss of power. Again, this issue could be resolved by the study of AAU cases not affected by AS in comparison with healthy controls.

## Behçet’s disease

Behçet’s disease (BD) is a chronic multisystemic inflammatory disorder characterized by recurrent uveitis, oral aphthae, arthritis, genital ulcerations, and skin inflammation. BD exists worldwide but has an ethnic predominance in countries along the ancient Silk Routes spanning from Asia to the Mediterranean basin, including China, Japan, Korea, and Turkey. Multiple genetic studies have provided evidence that genetic risk factors contribute to disease susceptibility. HLA-B*51 is the most strongly associated risk factor for BD, which was identified more than four decades ago and has been confirmed in multiple ethnic populations [25, 26]. With the exception of the strong association in the HLA region, multiple candidate gene association studies and GWAS have discovered non-HLA susceptibility loci and genes for BD. Herein, we review the progress of GWAS for BD thus far.

The first preliminary GWAS for BD was conducted in 2009, but this study was limited by mapping resolution and low detection power [27]. In 2010, two groups identified novel loci associated with BD by GWAS. Remmers et al. conducted a GWAS in 1,215 cases and 1,278 healthy controls from Turkey. They confirmed the association of BD with HLA-B*51 and identified associations at *IL10*, *IL23R-IL12RB2* and *CPLX1* [28]. At the same time, another group reported similar findings in different population. Mizuki et al. performed a GWAS in Japanese and also identified significant associations with SNPs in *IL23R-IL12RB2* and *IL10* [29]. Crucially, for the first time, these studies revealed significant SNPs associations in non-MHC regions. The first GWAS in Chinese population was conducted in 2012. Although the case sample size was small (149 cases and 951 controls in the discovery study), this study reported an association with SNPs in *STAT4* at genome-wide significance, and functional studies indicated its role in disease pathogenesis through the up-regulation of IL-17 production [30]. This association was intriguing, as STAT4 is part of the signalling pathway from IL-12 rather than IL-23. It is known that IL-17-inhibition with secukinumab is not effective in BD uveitis [31], and indeed cases of BD developing as an apparent complication of secukinumab treatment have been reported [32]. This, and the association of STAT4 with the disease, suggests that IL-12 and the Th1 pathway is more important in driving BD than is the IL-23 Th17 pathway. Subsequently, Kirino et al. performed imputation using previously genotyped data. and identified new susceptibility loci at *CCR1*, *STAT4*, *KLRC4* and *ERAP1* [33]. Additionally, this study discovered the evidence for interaction between HLA-B*51 and *ERAP1* [33]. This is of particular interest given the known interaction between *HLA-B27* and *ERAP1* variants in AS [34], and between *HLA-Cw6* and *ERAP1* variants in psoriasis [35]. In each case the interaction suggests that variations in antigenic peptide handling and processing by *ERAP* genes and HLA-B are involved in the disease pathogenesis. This study also demonstrated near genome-wide significant association of *IL12A* with BD, again consistent with the hypothesis that this is a T_H_1 driven disease. Another group conducted a GWAS for BD in Korean population. This study found a significant associations with SNPs in *GIMAP* gene cluster, which is involved in T-cell survival, but failed to replicate the association of the *IL23R-IL12RB2* and *IL10* loci [36]. In 2017, Takeuchi et al. using the immunogenetically targeted SNP microarray Immunochip studied the largest BD discovery collection thus far, comprising 1900 cases and 1,779 controls [37]. This study identified several new susceptibility loci reached genome-wide significance, *IL1A-IL1B*, *ADO-EGR2*, *CEBPB-PTPN1*, *IRF8*, *LACC1*, and *RIPK2*. A few loci previously reported for BD were also replicated, including HLA-B*51, *CCR1, ERAP1*, *FUT2, IL10, IL12A, IL23R-IL12RB2*, and *KLRC4* [37]. Pathway analysis showed these genes were involved in host defence, inflammation, and immune response. Moreover, this study increased the number of susceptibility loci known to be shared by BD and inflammatory bowel disease (IBD). Comparing among subgroups of IBD, Takeuchi et al. found higher genetic similarity of BD with Crohn’s disease than with ulcerative colitis [37].

GWAS is a powerful methodology to identify common variants associated with inflammatory diseases, but not suited for investigating the roles of rare or low-frequency variants with little or no surrounding linkage disequilibrium. Kirino et al. performed targeted resequencing of 21 genes, including 10 genes discovered by GWAS and 11 genes selected for their role in innate immunity, to compare the distribution of nonsynonymous variants with MAF < 1% in BD patients and healthy controls. This study using burden testing demonstrated suggestive association of *IL23R* and *TLR4* with BD, and demonstrated that the M694V *MEFV* polymorphism, which causes the autoinflammatory disease Familial Mediterranean Fever, is associated with BD [38].

## Vogt-Koyanagi-Harada disease

Vogt-Koyanagi-Harada (VKH) disease is a multisystemic autoimmune disorder characterized by bilateral panuveitis as well as neurological (aseptic meningitis), auditory (hearing loss, vertigo, tinnitus), and dermatological (vitiligo, poliosis, alopecia areata) manifestations. VKH disease is one of the most common form of uveitis in Asians, and is also prevalent in North Africans, Hispanics, and some natives of South America, whereas it is uncommon in Caucasians [39]. Gender differences have been observed in most studies, with females being likely to be affected than males, although this was no gender bias was observed in east Asian studies [40]. Although the exact pathogenesis of AAU remains unclear, recent genetic studies have enhanced our understanding of the disease aetiology. This review summarizes the main findings of genes associated with VKH disease.

The first strong association between HLA and VKH disease was discovered in 1981, namely HLA-DR4 and HLA-DRw53 [41]. The finding was since then confirmed in different ethnic populations. In addition to HLA-DR4 and HLA-DRw53, in certain populations, other HLA-antigens were also identified to be associated with VKH disease, such as HLA-DR1, HLA-DQw7, and HLA-DQ4 [42–44]. With advances in genotyping methodology, HLA can be defined not only by serologic typing, but also at the DNA level. Strong association of HLA-DQA1*0301, HLA-DQB1*0604, HLA-DRB1*0405 and HLA-DQB1*0401 with VKH disease have been observed in East Asian [43, 45, 46]. In contrast, HLA-DRB1*0404, DRB1*0102 and DRB1*0410 were associated with VKH disease in mestizo patients in Southern California [47], suggesting association of different HLA-antigens or alleles with VKH disease depending on the ethnic population. The complexity and magnitude of linkage disequilibrium across the MHC, and modest size of these studies has to date precluded definitive identification of the associated alleles.

In addition, various other non-HLA genes have also been shown to be associated at nominal levels of significance with VKH disease by candidate gene association studies. These studies focused on the association of certain important functional genes involved in either innate or adaptive immune response. These genes are related to Killer immunoglobulin-like receptors (KIR), complement components, pattern recognition receptors, cytokines, chemokines, transcription factors, cell activation and inhibition, apoptosis. The first GWAS for VKH, involving 744 Chinese cases and 2,009 healthy controls [48]. This study identified two new susceptibility loci associated with VKH disease reached genome-wide significance, *IL23R-C1orf141* (rs117633859) and *ADO-ZNF365-EGR2* (rs442309), it being of particular interest that these associations are shared by uveitis associated with both BD and AAU. These findings have recently been replicated in a Japanese case-control candidate gene association study [49]. Hou et al. also demonstrated association between HLA genes and VKH disease, showing the strongest association with SNP at *HLA-DRB1/DQA1* locus (rs3021304).

## Birdshot chorioretinopathy

Birdshot chorioretinopathy (BSCR) is a chronic, bilateral, autoimmune posterior uveitis typically affecting middle aged and elderly individuals of Caucasian origin. BSCR manifests as a severe progressive intraocular inflammation of the posterior eye segment, including vitritis, retinal vascular leakage, and cream-coloured spots at the level of the retinal pigment epithelium (RPE) or deeper retinal layers. As the spots scattered throughout the fundus that appear like birdshot from a shotgun, the first use of the term “birdshot chorioretinopathy” was introduced by Ryan and Maumenee in 1981 [50]. Unlike AAU, BD and VKH, birdshot chorioretinopathy is an uncommon form of uveitis, and is generally considered to be an isolated ocular disorder [51]. Although the strong association of BSCR and HLA-29 has been well recognised, the immunogenetic mechanisms of BSCR is still unclear, especially the genetic basis of BSCR. Here we summarize current knowledge in genetics of the disease, including the genetic association with HLA-A29 allele.

The association of HLA-A29 with BSCR was first reported almost four decades ago [52]. The prevalence of HLA-A29 in patients with BSCR is over 95% and the relative risk for developing disease in HLA-A29 positive individuals has been estimated to be as high as 224 [53]. Of note, as about 7% of the Caucasian population is HLA-A29 positive, so HLA-A29 is supportive of diagnosis, but not essential for diagnosis [54]. Consistently, the lack or low incidence of BSCR in certain Asian populations may be attributable to the low prevalence of the HLA-A29 allele [55]. HLA-A29 can be divided into more than 20 subtypes, but the most common subtypes in Caucasian population are HLA-A*29:01 and HLA-A*29:02, which have both been observed to have a strong association with BSCR [56]. HLA-A*29:01 and HLA-A*29.02 differ only by a single mutation (G376C/D102H), hence this does not appear to affect peptide binding [57, 58]. In addition, another much rarer allele, HLA-A*29:10, has also been incidentally reported in BSCR patients [59]. Although it was hypothesized that the association with HLA-A29 was in fact due to linkage disequilibrium with the actual casual genes, subsequent studies have found that short tandem repeats near *HLA-A* in patients revealed highly various haplotypes for HLA-A*29:01, A*29:02, and A*29:10, indicating that the *HLA-A29* gene itself confers risk to developing to BSCR [57, 59].

Recently, a GWAS of Northern Europeans also confirmed the significant association of HLA-A29 with BSCR. Kuiper et al. conducted a GWAS in individuals from Dutch and Spanish populations [60]. As expected, the strongest association signal was located within the HLA class I region for rs142115394. Intriguingly, fine-mapping the primary MHC association through HLA imputation revealed that HLA-A∗29:02 was the most significantly associated allele with BSCR, while HLA-A∗29:01 showed nominal association [60]. After adjusting for the HLA-A∗29:02 allele, no other classical HLA-A allele was seen to be significantly associated. These findings suggest that the HLA-A29 effect in BSCR can be primarily attributed to the HLA-A∗29:02 allele.

More importantly, this GWAS identified two novel susceptibility loci at 5q15 (rs7705093) near three members of the M1 family of aminopeptidases (*ERAP1*, *ERAP2* and *LNPEP*) and at 14q32.31 (rs150571175) in the *TECPR2* gene [60]. The *ERAP1/ERAP2* locus at chromosome 5q15 has strong linkage disequilibrium across it, and has variants with both strong effects on expression as well as on protein-coding and splice variation [61], making it challenging to determine primary associations. The authors then performed eQTL analysis to explore the biological relevance of the association at 5q15, and found the strongest impact was on *ERAP2* expression, suggesting *ERAP2* is the causal gene at this locus [60]. Functional analysis of ERAP2 protein expression and an independent replication study further confirmed the association of *ERAP2* with BSCR, suggesting a novel disease mechanism that affects peptide processing in the endoplasmic reticulum [60]. Recently, Sanz-Bravo et al. performed label-free quantitative mass spectrometry to characterize the effects of ERAP2 on the A*29:02-bound peptidome, which supported that the association of ERAP2 with BSCR is through its effects on peptide processing [62]. Of note, Alvarez-Navarro et al. conducted a study to investigate the influence of ERAP1 polymorphism on the amounts and features of HLA-A*29:02 ligands in human cells. The resulted showed that *ERAP1* polymorphism has a large influence, shaping the HLA-A*29:02 peptidome [63]. Future research on BSCR genetics with a larger sample size are needed to elucidate the roles of non-HLA genes, especially the *ERAP1*, *ERAP2* and *LNPEP* genes.

## Sarcoid uveitis

Sarcoidosis is a multi-system chronic inflammatory disease of unknown aetiology characterized by the formation of non-caseating granulomas in affected organs, most commonly the lungs, skin, lymphatics, and eyes. Sarcoidosis is one of the leading causes of inflammatory ocular disorder. The prevalence of ocular involvement in different ethnic population ranges from 12.9% (Turkish) to 79.2% (Japanese) of patients with sarcoidosis [64, 65]. Uveitis is the most frequent form of ocular manifestation and may affect up to 20–50% of sarcoidosis patients [66]. To date, multiple studies have demonstrated that genetic factors play an important role in sarcoidosis. Strong heritability has been demonstrated, with a study of twins from Denmark and Norway estimating heritability of sarcoidosis to be 66% [67]. HLA class II antigens, HLA-DRB1, and HLA-DQB1, have been identified to be associated with sarcoidosis [68, 69]. Moreover, GWAS and candidate gene studies have identified a few susceptibility loci for sarcoidosis, such as *BTNL2*, *NOTCH4*, *RAB23*, and *ANXA11* [70–72].

In contrast with these findings on overall sarcoidosis, there are few studies that have specifically investigated the association between genetic variants and sarcoid uveitis, with no GWAS yet published for sarcoid uveitis. Candidate gene studies have largely examined the associations between the known genes of overall sarcoidosis and sarcoid uveitis, mostly showing no significant differences in patients with sarcoid uveitis [73, 74]. Davoudi et al. identified SNPs in two sarcoid-related genes, *RAB23* and *ANXA11*, were associated with an increased risk of sarcoid uveitis based on the comparison of sarcoid uveitis versus sarcoid without uveitis [75]. Thompson et al. found that *CFH* Y402H polymorphism (rs1061170) was associated with sarcoid uveitis by comparing cases versus sarcoidosis-free controls [76]. *CFH* is known to be strongly associated with age-related macular degeneration. Other associations have been identified with SNPs in *HSPA1L* (also known as *HSP70-HOM*), *IL23R*, and *IL10*, although these have only been at nominal levels of significance [77–79]. Although these studies are all based on small sample size and lacking independent replication, the findings suggest the shared aetiology in sarcoid uveitis and other forms of uveitis. Future studies are necessary to reveal both specific associations and associations shared with overall sarcoidosis and other types of uveitis.

## Monogenic autoinflammatory disorders with uveitis

In addition to polygenic autoinflammatory diseases, a few monogenic autoinflammatory disorders can also cause uveitis. Herein, we introduce the genetic findings of Blau Syndrome and haploinsufficiency of A20.

### Blau Syndrome

Blau Syndrome (BS) is a rare monogenic autoinflammatory disease characterized by recurrent non-caseating granulomatous symmetric arthritis, dermatitis, and uveitis. In 1985, BS was firstly reported by Dr Blau, describing a dominant pedigree with paediatric onset of granulomatous uveitis, arthritis, and dermatitis [80]. BS is caused by mutations in the *NOD2* gene (also named *CARD15*), encoding nucleotide binding oligomerization domain containing 2, with autosomal dominant inheritance pattern [81]. *NOD2* genetic variants are associated with Crohn’s disease [82], and to a lesser extent with AS, psoriasis and ulcerative colitis [83]. Mutations in *NOD2* can also lead to early-onset sarcoidosis (EOS) [84]. Because of the striking clinical similarities and genetic background between BS and EOS, some researchers proposed that BS and EOS are the familial and sporadic forms, respectively, of the same disease [85]. On the other hand, some authors suggested to classify these patients as sporadic BS caused by de novo mutations in *NOD2* gene, using the term EOS to those patients without *NOD2* mutations [86]. Currently, more authors proposed that BS and EOS are the same non-caseating granulomatous inflammatory disease, respectively defined as the familial and sporadic forms [87, 88]. Although the prevalence and incidence of BS still remains unclear, more than 200 cases of BS have been reported worldwide by 2017 in the literature [89]. Reported mutations are mainly located in the NACHT domain, and might decrease the threshold for spontaneous oligomerization of NOD2. Among those *NOD2* mutations, R334W and R334Q are the most frequent mutations, suggesting codon 334 as a genetic hot spot for mutations [90]. Due to the complexity of clinical manifestations of BS, genetic analysis can significantly assist early diagnosis and treatment.

### Haploinsufficiency of A20

Haploinsufficiency of A20 (HA20) is a monogenic autoinflammatory disease with phenotypes resembling BD, caused by heterozygous mutations in *TNFAIP3* gene, which encodes the NF-κB regulatory protein A20. In 2016, HA20 was firstly reported by Zhou and colleagues, describing six unrelated families with early-onset systemic inflammation [91]. Using whole-exome sequencing and targeted sequencing, Zhou et al. identified three nonsense mutations and three frameshift mutations in *TNFAIP3* gene [91]. Since then, patients with HA20 were reported in different populations.

Because of the clinical similarities with BD, the majority of patients (>70%) were initially diagnosed or suspected of having BD [92]. Thus, it is of importance to recognise clinical characteristics that can assist to differentiate HA20 from BD. By comparison of clinical features between HA20 and BD, recent studies revealed a few specific features of HA20, including early-onset, autosomal dominant pedigree, recurrent fever, gastrointestinal involvement, and isolated anterior uveitis or retinal vasculitis [92–94]. Additionally, response to colchicine in HA20 is less frequent than in BD [95]. Thus, genetic testing of *TNFAIP3* gene is necessary to make molecular diagnosis for patients with HA20. Notably, as HA20 is a newly recognised autoinflammatory disease, prospective description of large cohort of patients with HA20 is essential to expand the knowledge in the future.

## Conclusions and future directions

Recent advances in molecular methods and genetic research methodology has greatly expanded our knowledge about the genetic basis of uveitis. Multiple studies have identified that genetic variants were associated with uveitis through influencing the expression of genes or the functions of gene products. These findings also have shed light on the disease mechanisms and clinical translational applications. Despite all these advances, research in this area is still facing considerable challenges.

A major reason is that uveitis is a diverse group of heterogeneous diseases characterized by different clinical findings involving both ocular and extraocular sites. Therefore, for most forms of uveitis the sample sizes studied to date by GWAS have only been large enough to robustly identify major genetic effects. Consequently, identified uveitis-related loci only account for a small fraction of the genetic basis of uveitis, indicating most of the causal genes of uveitis have yet to be discovered.

Another approach which has proven productive in other common heritable diseases is to investigate shared associations between diseases of overlapping aetiopathogenesis [83, 96]. As highlighted in this review, there is strong evidence both clinically and genetically of overlap between different forms of uveitis (Table 1) and other non-ocular diseases for which large genetic datasets are available, such as between sarcoidosis, inflammatory bowel disease, and AS. There is also suggestive evidence of shared genetic features between different forms of uveitis. In Table 1, most of these SNPs were in linkage disequilibrium and the risk alleles were in the same direction. For examples, in the *ADO-EGR2* Locus, rs224127 of BD was in very strong linkage disequilibrium with rs442309 of VKH (D’ = 1; R^2^ = 0.867), and showed the consistent direction of effect (OR > 1). In the CFB locus, the same SNP (rs1048709) for AAU and VKH, also showed the same direction of effect (OR > 1). Searches for pleiotropic associations across these disease-types would seem likely to be productive in identifying novel associations with uveitis.

**Table 1:** Overlaps between genetic associations (achieving genome-wide significance) across the different forms of uveitis.

Locus	Chr	AAU	BD	VKH	BSCR	Sarcoid uveitis
*ADO-EGR2*	10		rs224127/A OR = 1.27 [99]	rs442309/T OR = 1.37 [48]		
*CFB*	6	rs1048709/A OR = 1.99 [21]		rs1048709/A OR = 1.49 [100]		
*CFH*	1	rs800292/G OR = 2.10 [101]				rs1061170/C OR = 1.72 [76]
*ERAP1-ERAP2-LNPEP*	5	rs2032890/A OR = 1.51 [102]	rs17482078/TT OR = 4.56 [33]		rs7705093/T OR = 2.3 [60]	
*IL10-IL19*	1	rs17351243/A OR = 1.24 [102]	rs1518111/A OR = 1.41 [28]			rs1800871/TT OR = 1.67 [79]
*IL23R*	1	rs79755370/C OR = 1.80 [102]	rs924080/A OR = 1.31 [28]	rs117633859/G OR = 1.82 [48]		rs11465804/G OR = 0.11 [78]
*STAT4*	2		rs7574070/A OR = 1.27 [33]	rs7574865/T NA [103]		
*TRAF5*	1	rs12569232/C OR = 0.34 [104]	rs12569232/G OR = 1.48 [105]	rs12569232/C OR = 0.44 [105]		

The step of translating GWAS findings to functionally relevant understanding has been greatly aided in recent times by the development of Mendelian randomisation approaches, using GWAS data from disease studies to investigate potential causative relationships between diseases, traits and other measures, including epigenetic, immunological, proteomic and transcriptomic data [97, 98]. Very few studies in uveitis genetics have employed this approach, despite in some diseases sufficiently large datasets being available to employ it. This would seem a potentially valuable avenue for future research in this field.

Ultimately though, much larger sample sizes need to be studied to properly dissect the genetics of these conditions. Given the huge unmet need for therapies for these vision- and in some cases life-threatening diseases, international collaboration to achieve the requisite sample sizes appears called for. With genotyping costs having now fallen to quite low levels, the limiting factor in performing these studies is case recruitment. Given that most of these diseases are managed in specialised units in major hospitals, this is surely surmountable. If sufficiently large case cohorts are recruited for genetic studies, the record so far both in immune-mediated diseases overall, and in uveitis itself, shows that it is likely that information gleaned from these hypothesis-free studies will provide valuable information about the pathogenesis of these diseases, and point to potential treatments.

## Data Availability

This article contains no original research data.

## References

[1] Miserocchi E, Fogliato G, Modorati G, Bandello F (2013). Review on the worldwide epidemiology of uveitis. Eur J Ophthalmol.

[2] Yang P, Zhang Z, Zhou H, Li B, Huang X, Gao Y (2005). Clinical patterns and characteristics of uveitis in a tertiary center for uveitis in China. Curr eye Res.

[3] Martin TM, Rosenbaum JT (2005). Genetics in uveitis. Int Ophthalmol Clin.

[4] Brewerton DA, Caffrey M, Nicholls A, Walters D, James DC (1973). Acute anterior uveitis and HL-A 27. Lancet.

[5] Visscher PM, Wray NR, Zhang Q, Sklar P, McCarthy MI, Brown MA (2017). 10 years of GWAS discovery: biology, function, and translation. Am J Hum Genet.

[6] Wakefield D, Montanaro A, McCluskey P (1991). Acute anterior uveitis and HLA-B27. Surv Ophthalmol.

[7] Monnet D, Breban M, Hudry C, Dougados M, Brezin AP (2004). Ophthalmic findings and frequency of extraocular manifestations in patients with HLA-B27 uveitis: a study of 175 cases. Ophthalmology.

[8] Wang Y, Huang XF, Yang MM, Cai WJ, Zheng MQ, Mao G (2014). CFI-rs7356506 is a genetic protective factor for acute anterior uveitis in Chinese patients. Br J Ophthalmol.

[9] Derhaag PJ, Linssen A, Broekema N, de Waal LP, Feltkamp TE (1988). A familial study of the inheritance of HLA-B27-positive acute anterior uveitis. Am J Ophthalmol.

[10] Huang XF, Li Z, De Guzman E, Robinson P, Gensler L, Ward MM (2020). Genomewide association study of acute anterior uveitis identifies new susceptibility loci. Investig Ophthalmol Vis Sci.

[11] Lan C, Tam PO, Chiang SW, Chan CK, Luk FO, Lee GK (2009). Manganese superoxide dismutase and chemokine genes polymorphisms in Chinese patients with anterior uveitis. Investig Ophthalmol Vis Sci.

[12] Robinson PC, Claushuis TA, Cortes A, Martin TM, Evans DM, Leo P (2015). Genetic dissection of acute anterior uveitis reveals similarities and differences in associations observed with ankylosing spondylitis. Arthritis Rheumatol.

[13] Robinson PC, Leo PJ, Pointon JJ, Harris J, Cremin K, Bradbury LA (2016). The genetic associations of acute anterior uveitis and their overlap with the genetics of ankylosing spondylitis. Genes Immun.

[14] Derakhshan MH, Dean L, Jones GT, Siebert S, Gaffney K. Predictors of extra-articular manifestations in axial spondyloarthritis and their influence on TNF-inhibitor prescribing patterns: results from the British Society for Rheumatology Biologics Register in Ankylosing Spondylitis. RMD open. 2020;6. 10.1136/rmdopen-2020-001206 PMC7425116 32641447

[15] Cortes A, Brown MA (2011). Promise and pitfalls of the Immunochip. Arthritis Res Ther.

[16] Reveille JD, Zhou X, Lee M, Weisman MH, Yi L, Gensler LS (2019). HLA class I and II alleles in susceptibility to ankylosing spondylitis. Ann Rheum Dis.

[17] Yang MM, Lai TY, Luk FO, Pang CP (2014). The roles of genetic factors in uveitis and their clinical significance. Retina.

[18] Huang XF, Chi W, Lin D, Dai ML, Wang YL, Yang YM (2018). Association of IL33 and IL1RAP polymorphisms with acute anterior uveitis. Curr Mol Med.

[19] Li H, Hou S, Yu H, Zheng M, Zhang L, Zhang J (2015). Association of genetic variations in TNFSF15 with acute anterior uveitis in Chinese Han. Investigative Ophthalmol Vis Sci.

[20] Yang MM, Lai TY, Tam PO, Chiang SW, Ng TK, Rong SS (2013). Association of CFH and SERPING1 polymorphisms with anterior uveitis. Br J Ophthalmol.

[21] Yang MM, Lai TY, Tam PO, Chiang SW, Ng TK, Liu K (2012). Association of C2 and CFB polymorphisms with anterior uveitis. Investig Ophthalmol Vis Sci.

[22] Huang XF, Lin D, Lin KH, Lee SH, Xia X, Yang YM (2018). Genotype-phenotype association study reveals CFI-Rs13104777 to be a protective genetic marker against acute anterior uveitis. Ocul Immunol Inflamm.

[23] Huang XF, Wang Y, Li FF, Lin D, Dai ML, Wang QF (2016). CFHR2-rs2986127 as a genetic protective marker for acute anterior uveitis in Chinese patients. J gene Med.

[24] Li Z, Wu X, Leo PJ, De Guzman E, Akkoc N, Breban M (2021). Polygenic Risk Scores have high diagnostic capacity in ankylosing spondylitis. Ann Rheum Dis.

[25] Ohno S, Aoki K, Sugiura S, Nakayama E, Itakura K, Aizawa M (1973). Letter: HL-A5 and Behcet’s disease. Lancet.

[26] Gul A, Ohno S (2012). HLA-B*51 and Behcet Disease. Ocul Immunol Inflamm.

[27] Fei Y, Webb R, Cobb BL, Direskeneli H, Saruhan-Direskeneli G, Sawalha AH (2009). Identification of novel genetic susceptibility loci for Behcet’s disease using a genome-wide association study. Arthritis Res Ther.

[28] Remmers EF, Cosan F, Kirino Y, Ombrello MJ, Abaci N, Satorius C (2010). Genome-wide association study identifies variants in the MHC class I, IL10, and IL23R-IL12RB2 regions associated with Behcet’s disease. Nat Genet.

[29] Mizuki N, Meguro A, Ota M, Ohno S, Shiota T, Kawagoe T (2010). Genome-wide association studies identify IL23R-IL12RB2 and IL10 as Behcet’s disease susceptibility loci. Nat Genet.

[30] Hou S, Yang Z, Du L, Jiang Z, Shu Q, Chen Y (2012). Identification of a susceptibility locus in STAT4 for Behcet’s disease in Han Chinese in a genome-wide association study. Arthritis Rheumatism.

[31] Dick AD, Tugal-Tutkun I, Foster S, Zierhut M, Melissa Liew SH, Bezlyak V (2013). Secukinumab in the treatment of noninfectious uveitis: results of three randomized, controlled clinical trials. Ophthalmology.

[32] Dincses E, Yurttas B, Esatoglu SN, Melikoglu M, Hamuryudan V, Seyahi E (2019). Secukinumab induced Behcet’s syndrome: a report of two cases. Oxf Med Case Rep.

[33] Kirino Y, Bertsias G, Ishigatsubo Y, Mizuki N, Tugal-Tutkun I, Seyahi E (2013). Genome-wide association analysis identifies new susceptibility loci for Behcet’s disease and epistasis between HLA-B*51 and ERAP1. Nat Genet.

[34] Evans DM, Spencer CC, Pointon JJ, Su Z, Harvey D, Kochan G (2011). Interaction between ERAP1 and HLA-B27 in ankylosing spondylitis implicates peptide handling in the mechanism for HLA-B27 in disease susceptibility. Nat Genet.

[35] Strange A, Capon F, Spencer CC, Knight J, Genetic Analysis of Psoriasis C, the Wellcome Trust Case Control C, (2010). A genome-wide association study identifies new psoriasis susceptibility loci and an interaction between HLA-C and ERAP1. Nat Genet.

[36] Lee YJ, Horie Y, Wallace GR, Choi YS, Park JA, Choi JY (2013). Genome-wide association study identifies GIMAP as a novel susceptibility locus for Behcet’s disease. Ann Rheum Dis.

[37] Takeuchi M, Mizuki N, Meguro A, Ombrello MJ, Kirino Y, Satorius C (2017). Dense genotyping of immune-related loci implicates host responses to microbial exposure in Behcet’s disease susceptibility. Nat Genet.

[38] Kirino Y, Zhou Q, Ishigatsubo Y, Mizuki N, Tugal-Tutkun I, Seyahi E (2013). Targeted resequencing implicates the familial Mediterranean fever gene MEFV and the toll-like receptor 4 gene TLR4 in Behcet disease. Proc Natl Acad Sci USA.

[39] Du L, Kijlstra A, Yang P (2016). Vogt-Koyanagi-Harada disease: novel insights into pathophysiology, diagnosis and treatment. Prog retinal eye Res.

[40] Yang P, Ren Y, Li B, Fang W, Meng Q, Kijlstra A (2007). Clinical characteristics of Vogt-Koyanagi-Harada syndrome in Chinese patients. Ophthalmology.

[41] Ohno S (1981). Immunological aspects of Behcet’s and Vogt-Koyanagi-Harada’s diseases. Trans Ophthalmol Soc U Kingd.

[42] Weisz JM, Holland GN, Roer LN, Park MS, Yuge AJ, Moorthy RS (1995). Association between Vogt-Koyanagi-Harada syndrome and HLA-DR1 and -DR4 in Hispanic patients living in southern California. Ophthalmology.

[43] Islam SM, Numaga J, Fujino Y, Hirata R, Matsuki K, Maeda H (1994). HLA class II genes in Vogt-Koyanagi-Harada disease. Investig Ophthalmol Vis Sci.

[44] Zhang XY, Wang XM, Hu TS (1992). Profiling human leukocyte antigens in Vogt-Koyanagi-Harada syndrome. Am J Ophthalmol.

[45] Shindo Y, Ohno S, Yamamoto T, Nakamura S, Inoko H (1994). Complete association of the HLA-DRB1*04 and -DQB1*04 alleles with Vogt-Koyanagi-Harada’s disease. Hum Immunol.

[46] Kim MH, Seong MC, Kwak NH, Yoo JS, Huh W, Kim TG (2000). Association of HLA with Vogt-Koyanagi-Harada syndrome in Koreans. Am J Ophthalmol.

[47] Levinson RD, See RF, Rajalingam R, Reed EF, Park MS, Rao NA (2004). HLA-DRB1 and -DQB1 alleles in mestizo patients with Vogt-Koyanagi-Harada’s disease in Southern California. Hum Immunol.

[48] Hou S, Du L, Lei B, Pang CP, Zhang M, Zhuang W (2014). Genome-wide association analysis of Vogt-Koyanagi-Harada syndrome identifies two new susceptibility loci at 1p31.2 and 10q21.3. Nat Genet.

[49] Sakono T, Meguro A, Takeuchi M, Yamane T, Teshigawara T, Kitaichi N (2020). Variants in IL23R-C1orf141 and ADO-ZNF365-EGR2 are associated with susceptibility to Vogt-Koyanagi-Harada disease in Japanese population. PloS one.

[50] Ryan SJ, Maumenee AE (1980). Birdshot retinochoroidopathy. Am J Ophthalmol.

[51] Pagnoux C, Mahr A, Aouba A, Berezne A, Monnet D, Cohen P (2010). Extraocular manifestations of birdshot chorioretinopathy in 118 French patients. Presse Med.

[52] Nussenblatt RB, Mittal KK, Ryan S, Green WR, Maumenee AE (1982). Birdshot retinochoroidopathy associated with HLA-A29 antigen and immune responsiveness to retinal S-antigen. Am J Ophthalmol.

[53] Brezin AP, Monnet D, Cohen JH, Levinson RD (2011). HLA-A29 and birdshot chorioretinopathy. Ocul Immunol Inflamm.

[54] Levinson RD, Brezin A, Rothova A, Accorinti M, Holland GN (2006). Research criteria for the diagnosis of birdshot chorioretinopathy: results of an international consensus conference. Am J Ophthalmol.

[55] Saito S, Ota S, Yamada E, Inoko H, Ota M (2000). Allele frequencies and haplotypic associations defined by allelic DNA typing at HLA class I and class II loci in the Japanese population. Tissue Antigens.

[56] Levinson RD, Rajalingam R, Park MS, Reed EF, Gjertson DW, Kappel PJ (2004). Human leukocyte antigen A29 subtypes associated with birdshot retinochoroidopathy. Am J Ophthalmol.

[57] Donvito B, Monnet D, Tabary T, Delair E, Vittier M, Reveil B (2005). Different HLA class IA region complotypes for HLA-A29.2 and -A29.1 antigens, identical in birdshot retinochoroidopathy patients or healthy individuals. Investig Ophthalmol Vis Sci.

[58] Minos E, Barry RJ, Southworth S, Folkard A, Murray PI, Duker JS (2016). Birdshot chorioretinopathy: current knowledge and new concepts in pathophysiology, diagnosis, monitoring and treatment. Orphanet J Rare Dis.

[59] Donvito B, Monnet D, Tabary T, Delair E, Vittier M, Reveil B (2010). A new HLA extended haplotype containing the A*2910 allele in birdshot retinochoroidopathy: susceptibility narrowed to the HLA molecule itself. Investig Ophthalmol Vis Sci.

[60] Kuiper JJ, Van Setten J, Ripke S, Van TSR, Mulder F, Missotten T (2014). A genome-wide association study identifies a functional ERAP2 haplotype associated with birdshot chorioretinopathy. Hum Mol Genet.

[61] Hanson AL, Cuddihy T, Haynes K, Loo D, Morton CJ, Oppermann U (2018). Genetic variants in ERAP1 and ERAP2 associated with immune-mediated diseases influence protein expression and the isoform profile. Arthritis Rheumatol.

[62] Sanz-Bravo A, Martin-Esteban A, Kuiper JJW, Garcia-Peydro M, Barnea E, Admon A (2018). Allele-specific alterations in the peptidome underlie the joint association of HLA-A*29:02 and Endoplasmic Reticulum Aminopeptidase 2 (ERAP2) with Birdshot Chorioretinopathy. Mol Cell Proteom: MCP.

[63] Alvarez-Navarro C, Martin-Esteban A, Barnea E, Admon A, Lopez de Castro JA (2015). Endoplasmic Reticulum Aminopeptidase 1 (ERAP1) Polymorphism relevant to inflammatory disease shapes the peptidome of the birdshot chorioretinopathy-associated HLA-A*29:02 antigen. Mol Cell Proteom: MCP.

[64] Atmaca LS, Atmaca-Sonmez P, Idil A, Kumbasar OO, Celik G (2009). Ocular involvement in sarcoidosis. Ocul Immunol Inflamm.

[65] Ohara K, Okubo A, Sasaki H, Kamata K (1992). Intraocular manifestations of systemic sarcoidosis. Jpn J Ophthalmol.

[66] Jamilloux Y, Kodjikian L, Broussolle C, Seve P (2014). Sarcoidosis and uveitis. Autoimmun Rev.

[67] Sverrild A, Backer V, Kyvik KO, Kaprio J, Milman N, Svendsen CB (2008). Heredity in sarcoidosis: a registry-based twin study. Thorax.

[68] Rossman MD, Thompson B, Frederick M, Maliarik M, Iannuzzi MC, Rybicki BA (2003). HLA-DRB1*1101: a significant risk factor for sarcoidosis in blacks and whites. Am J Hum Genet.

[69] Iannuzzi MC, Maliarik MJ, Poisson LM, Rybicki BA (2003). Sarcoidosis susceptibility and resistance HLA-DQB1 alleles in African Americans. Am J Respir Crit care Med.

[70] Hofmann S, Franke A, Fischer A, Jacobs G, Nothnagel M, Gaede KI (2008). Genome-wide association study identifies ANXA11 as a new susceptibility locus for sarcoidosis. Nat Genet.

[71] Hofmann S, Fischer A, Till A, Muller-Quernheim J, Hasler R, Franke A (2011). A genome-wide association study reveals evidence of association with sarcoidosis at 6p12.1. Eur Respir J.

[72] Adrianto I, Lin CP, Hale JJ, Levin AM, Datta I, Parker R (2012). Genome-wide association study of African and European Americans implicates multiple shared and ethnic specific loci in sarcoidosis susceptibility. PloS one.

[73] Suzuki H, Ota M, Meguro A, Katsuyama Y, Kawagoe T, Ishihara M (2012). Genetic characterization and susceptibility for sarcoidosis in Japanese patients: risk factors of BTNL2 gene polymorphisms and HLA class II alleles. Investig Ophthalmol Vis Sci.

[74] Chaperon M, Pacheco Y, Maucort-Boulch D, Iwaz J, Perard L, Broussolle C (2019). BTNL2 gene polymorphism and sarcoid uveitis. Br J Ophthalmol.

[75] Davoudi S, Chang VS, Navarro-Gomez D, Stanwyck LK, Sevgi DD, Papavasileiou E (2018). Association of genetic variants in RAB23 and ANXA11 with uveitis in sarcoidosis. Mol Vis.

[76] Thompson IA, Liu B, Sen HN, Jiao X, Katamay R, Li Z (2013). Association of complement factor H tyrosine 402 histidine genotype with posterior involvement in sarcoid-related uveitis. Am J Ophthalmol.

[77] Spagnolo P, Sato H, Marshall SE, Antoniou KM, Ahmad T, Wells AU (2007). Association between heat shock protein 70/Hom genetic polymorphisms and uveitis in patients with sarcoidosis. Investig Ophthalmol Vis Sci.

[78] Kim HS, Choi D, Lim LL, Allada G, Smith JR, Austin CR (2011). Association of interleukin 23 receptor gene with sarcoidosis. Dis Mark.

[79] Kuroda H, Saijo Y, Fujiuchi S, Takeda H, Ohsaki Y, Hasebe N (2013). Relationship between cytokine single nucleotide polymorphisms and sarcoidosis among Japanese subjects. Sarcoidosis Vasculitis Diffus lung Dis: Off J WASOG.

[80] Blau EB (1985). Familial granulomatous arthritis, iritis, and rash. J Pediatr.

[81] Miceli-Richard C, Lesage S, Rybojad M, Prieur AM, Manouvrier-Hanu S, Hafner R (2001). CARD15 mutations in Blau syndrome. Nat Genet.

[82] Hugot JP, Chamaillard M, Zouali H, Lesage S, Cezard JP, Belaiche J (2001). Association of NOD2 leucine-rich repeat variants with susceptibility to Crohn’s disease. Nature.

[83] Ellinghaus D, Jostins L, Spain SL, Cortes A, Bethune J, Han B (2016). Analysis of five chronic inflammatory diseases identifies 27 new associations and highlights disease-specific patterns at shared loci. Nat Genet.

[84] Kanazawa N, Matsushima S, Kambe N, Tachibana T, Nagai S, Miyachi Y (2004). Presence of a sporadic case of systemic granulomatosis syndrome with a CARD15 mutation. J Investig Dermatol.

[85] Rose CD, Doyle TM, McIlvain-Simpson G, Coffman JE, Rosenbaum JT, Davey MP (2005). Blau syndrome mutation of CARD15/NOD2 in sporadic early onset granulomatous arthritis. J Rheumatol.

[86] Milman N, Ursin K, Rodevand E, Nielsen FC, Hansen TV (2009). A novel mutation in the NOD2 gene associated with Blau syndrome: a Norwegian family with four affected members. Scand J Rheumatol.

[87] Caso F, Costa L, Rigante D, Vitale A, Cimaz R, Lucherini OM (2014). Caveats and truths in genetic, clinical, autoimmune and autoinflammatory issues in Blau syndrome and early onset sarcoidosis. Autoimmun Rev.

[88] Caso F, Galozzi P, Costa L, Sfriso P, Cantarini L, Punzi L (2015). Autoinflammatory granulomatous diseases: from Blau syndrome and early-onset sarcoidosis to NOD2-mediated disease and Crohn’s disease. RMD open.

[89] Li C, Zhang J, Li S, Han T, Kuang W, Zhou Y (2017). Gene mutations and clinical phenotypes in Chinese children with Blau syndrome. Sci China Life Sci.

[90] Matsuda T, Kambe N, Ueki Y, Kanazawa N, Izawa K, Honda Y (2020). Clinical characteristics and treatment of 50 cases of Blau syndrome in Japan confirmed by genetic analysis of the NOD2 mutation. Ann Rheum Dis.

[91] Zhou Q, Wang H, Schwartz DM, Stoffels M, Park YH, Zhang Y (2016). Loss-of-function mutations in TNFAIP3 leading to A20 haploinsufficiency cause an early-onset autoinflammatory disease. Nat Genet.

[92] Aeschlimann FA, Batu ED, Canna SW, Go E, Gul A, Hoffmann P (2018). A20 haploinsufficiency (HA20): clinical phenotypes and disease course of patients with a newly recognised NF-kB-mediated autoinflammatory disease. Ann Rheum Dis.

[93] Tsuchida N, Kirino Y, Soejima Y, Onodera M, Arai K, Tamura E (2019). Haploinsufficiency of A20 caused by a novel nonsense variant or entire deletion of TNFAIP3 is clinically distinct from Behcet’s disease. Arthritis Res Ther.

[94] Kone-Paut I, Georgin-Laviallec S, Galeotti C, Rossi-Semerano L, Hentgen V, Savey L (2019). New data in causes of autoinflammatory diseases. Jt Bone Spine.

[95] Berteau F, Rouviere B, Delluc A, Nau A, Le Berre R, Sarrabay G (2018). Autosomic dominant familial Behcet disease and haploinsufficiency A20: a review of the literature. Autoimmun Rev.

[96] Huang J, Johnson AD, O’Donnell CJ (2011). PRIMe: a method for characterization and evaluation of pleiotropic regions from multiple genome-wide association studies. Bioinformatics.

[97] Evans DM, Davey, Smith G (2015). Mendelian randomization: new applications in the coming age of hypothesis-free causality. Annu Rev Genomics Hum Genet.

[98] Zhu Z, Zheng Z, Zhang F, Wu Y, Trzaskowski M, Maier R (2018). Causal associations between risk factors and common diseases inferred from GWAS summary data. Nat Commun.

[99] Wu P, Du L, Hou S, Su G, Yang L, Hu J (2018). Association of LACC1, CEBPB-PTPN1, RIPK2 and ADO-EGR2 with ocular Behcet’s disease in a Chinese Han population. Br J Ophthalmol.

[100] Yang M, Fan JJ, Wang J, Zhao Y, Teng Y, Liu P (2016). Association of the C2-CFB locus with non-infectious uveitis, specifically predisposed to Vogt-Koyanagi-Harada disease. Immunol Res.

[101] Yang MM, Lai TY, Tam PO, Chiang SW, Chan CK, Luk FO (2011). CFH 184G as a genetic risk marker for anterior uveitis in Chinese females. Mol Vis.

[102] Robinson PC, Claushuis TA, Cortes A, Martin TM, Evans DM, Leo P (2015). Genetic dissection of acute anterior uveitis reveals similarities and differences in associations observed with ankylosing spondylitis. Arthritis Rheumatol.

[103] Hu K, Yang P, Jiang Z, Hou S, Du L, Li F (2010). STAT4 polymorphism in a Chinese Han population with Vogt-Koyanagi-Harada syndrome and Behcet’s disease. Hum Immunol.

[104] Xiang Q, Chen L, Fang J, Hou S, Wei L, Bai L (2013). TNF receptor-associated factor 5 gene confers genetic predisposition to acute anterior uveitis and pediatric uveitis. Arthritis Res Ther.

[105] Xiang Q, Chen L, Hou S, Fang J, Zhou Y, Bai L (2014). TRAF5 and TRAF3IP2 gene polymorphisms are associated with Behcet’s disease and Vogt-Koyanagi-Harada syndrome: a case-control study. PloS One.

